# Delivering fracture prevention services to rural US veterans through telemedicine: a process evaluation

**DOI:** 10.1007/s11657-021-00882-0

**Published:** 2021-02-10

**Authors:** Karla L. Miller, Melissa J. Steffen, Kimberly D. McCoy, Grant Cannon, Aaron T. Seaman, Zachary L. Anderson, Shardool Patel, Janiel Green, Shylo Wardyn, Samantha L. Solimeo

**Affiliations:** 1 VA Office of Rural Health, Veterans Rural Health Resource Center-Salt Lake City (VRHRC-SLC), Salt Lake City, UT USA; 2 Department of Internal Medicine, Rheumatology Section, Veterans Affairs Salt Lake City Health Care System, Salt Lake City, UT USA; 3 Division of Rheumatology, University of Utah School of Medicine, Salt Lake City, UT USA; 4 VA Office of Rural Health, Veterans Rural Health Resource Center-Iowa City (VRHRC-IC), Salt Lake City, UT USA; 5 Comprehensive Access & Delivery Research and Evaluation (CADRE), Primary Care Analytics Team Iowa City (PCAT-IC), Department of Veterans Affairs, CADRE, Iowa City VA HCS, Research 152, 601 Highway 6 West, Iowa City, IA 52246 USA; 6 Division of Genera l Internal Medicine, Department of Internal Medicine, University of Iowa Carver College of Medicine, 200 Hawkins Drive, 52242 Iowa City, IA USA; 7 Department of Anesthesiology, Veterans Affairs Salt Lake City Health Care System, Salt Lake City, UT USA; 8 Division of Epidemiology, Department of Internal Medicine, University of Utah School of Medicine, Salt Lake City, UT USA; 9 Veterans Affairs Salt Lake City Health Care System, Salt Lake City, UT USA

**Keywords:** Primary prevention, Fracture, Telehealth, Rural

## Abstract

***Summary*:**

An informatics-driven population bone health clinic was implemented to identify, screen, and treat rural US Veterans at risk for osteoporosis. We report the results of our implementation process evaluation which demonstrated BHT to be a feasible telehealth model for delivering preventative osteoporosis services in this setting.

**Purpose:**

An established and growing quality gap in osteoporosis evaluation and treatment of at-risk patients has yet to be met with corresponding clinical care models addressing osteoporosis primary prevention. The rural bone health tea m (BHT) was implemented to identify, screen, and treat rural Veterans lacking evidence of bone health care and we conducted a process evaluation to understand BHT implementation feasibility.

**Methods:**

For this evaluation, we defined the primary outcome as the number of Veterans evaluated with DXA and a secondary outcome as the number of Veterans who initiated prescription therapy to reduce fracture risk. Outcomes were measured over a 15-month period and analyzed descriptively. Qualitative data to understand successful implementation were collected concurrently by conducting interviews with clinical personnel interacting with BHT and BHT staff and observations of BHT implementation processes at three site visits using the Promoting Action on Research Implementation in Health Services (PARIHS) framework.

**Results:**

Of 4500 at-risk, rural Veterans offered osteoporosis screening, 1081 (24%) completed screening, and of these, 37% had normal bone density, 48% osteopenia, and 15% osteoporosis. Among Veterans with pharmacotherapy indications, 90% initiated therapy. Qualitative analyses identified barriers of rural geography, rural population characteristics, and the infrastructural resource requirement. Data infrastructure, evidence base for care delivery, stakeholder buy-in, formal and informal facilitator engagement, and focus on teamwork were identified as facilitators of implementation success.

**Conclusion:**

The BHT is a feasible population telehealth model for delivering preventative osteoporosis care to rural Veterans.

## Introduction

Osteoporosis underdiagnosis and undertreatment contribute to fractures and mortality among older adults, despite validated risk stratification tools to identify patients at risk of fracture [1] and medications to reduce this risk [2]. Prior research has identified many contributing factors to the low rates of osteoporosis screening and treatment, including differing clinical guidelines for identifying patients with indication for dual-energy X-ray absorptiometry (DXA) screening of bone mineral density (BMD) [3–6]; reimbursement policies; limited time during clinic visits to complete fracture risk assessment using existing stratification tools; and limited provider understanding of risk [7, 8]. Organizational and education barriers may be overcome by using an informatics approach to identify patients with un-assessed fracture risk on the basis of their electronic health record data. However, few healthcare systems have access to sufficient depth and breadth of clinical information to adopt a population health approach to screening.

The Veterans Health Administration (VHA) uses a national electronic health record (EHR) which integrates inpatient, outpatient, sociodemographic, and pharmacy data and is uniquely positioned to advance the science and quality of osteoporosis screening. Further, in comparison to the private sector, osteoporosis care delivery innovations in the VHA draw from a range of available implementation resources. In addition to the EHR, programs can build upon access to free or low cost DXA for both men and women, low cost oral medications provided by mail, access to injection medications as indicated through nonformulary requests, and access to allied health practitioners without excessive administrative justification (e.g., physical therapy for balance training, occupational therapy for home safety evaluations, or dietician for nutritional counseling). Despite these advantages, rates of DXA and medication use among Veterans with fracture risk are comparable to those in the private sector, with rural patients having lower rate of care than their urban counterparts [9–11].

In this context, the VA Office of Rural Health, Veterans Rural Health Resource Center-Salt Lake City (VRHRC- SLC) supported the implementation and expansion of previously piloted osteoporosis telehealth clinic, beginning in 2017 [12]. Known as the Rural Bone Health Team (BHT), the telehealth clinic co -manages osteoporosis care with a Veteran’s primary care provider (PCP) for Veterans served by PCPs located at community-based outpatient clinics in Utah, Wyoming, and Colorado. The BHT assumed clinical ownership of Veteran osteoporosis risk identification, bone health evaluation, and management, while limiting workload offset to PCPs. Co-management was achieved by using existing telehealth technology (i.e., BHT-initiated telephone consults with rural primary care patients at elevated risk of fracture) and EHR capabilities, facilitated by a care coordination agreement with primary care. In parallel to the clinical team supported by VRHRC-SLC (authors KM, GC, JG, ZLA, SP), the Veterans Rural Health Resource Center–Iowa City (VRHRC-IC) funded an evaluation team (authors SLS, ATS, MS, SW) to study clinic implementation. In this manuscript, we report the results of a process evaluation conducted jointly by the clinical and evaluation teams. We first describe BHT’s structure, enrollment criteria, and clinical processes. We then report the results of a multi-method process evaluation conducted to illustrate the feasibility of and challenges inherent to implementing a telehealth osteoporosis clinic to deliver population-based bone health care in a rural region of the USA.

### Rural bone health team’s structure, enrollment criteria, and clinical processes

The BHT comprises program support assistants (PSA), clinical nurses with expanded scopes of practice, advanced practice providers (APP, i.e., physician assistant and clinical pharmacist), and a supervising rheumatologist with expertise in osteoporosis [12]. The PSAs provide administrative support such as preparing and monitoring clinic enrollment materials to identified Veterans; mailing communications from APPs or RNs regarding screening, diagnosis, and treatment; monitoring DXA results obtained in the community; and fielding incoming patient inquiries. The diagnosis of normal, low bone density (i.e., osteopenia), and osteoporosis by measurement of BMD using DXA are based on the World Health Organization diagnostic classifications [4, 6, 13], in addition to clinical diagnosis by low-trauma hip or vertebral fracture in adulthood. The RNs work under a n evidence-based clinical protocol and are responsible for initial telephone contact with all Veterans who accept clinic enrollment; reviewing self-reported clinical information; assessing and counseling for age-appropriate calcium/vitamin D intake, smoking and alcohol cessation; assessing indications for fall risk and home safety; placing orders for DXA, physical therapy (PT), and occupational therapy (OT) home safety evaluation; and calculating FRAX® in all Veterans with a radiologic diagnosis of osteopenia [14, 15]. RNs communicate normal or osteopenia with low risk results to the Veteran with instructions on next DXA and stratify patients with high fracture risk for further evaluation by an APP.

**Fig. 1:**
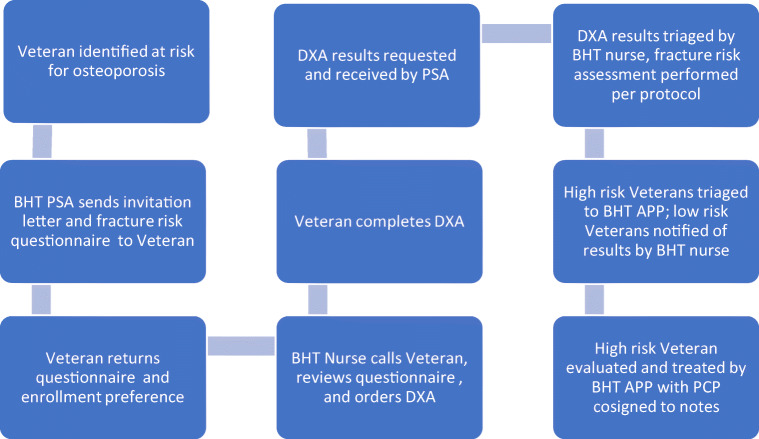
Rural BHT clinical process workflow

The APPs then perform clinical evaluation and make treatment recommendations for patients found to have osteoporosis by DXA, osteoporosis by fragility fracture of the hip or spine, or osteopenia with high risk for fracture by FRAX®. Figure 1 illustrates the clinical process workflow of the Rural BHT.

Enrollment criteria address both fracture risk and the rural disparity in care. Eligibility criteria were as follows: rural residence (defined using census tract Rural Urban Commuting Area s [16]), evidence of regular VHA primary care (i.e., having an assigned VA primary care provider), renal sufficiency for treatment (i.e., creatinine clearance ≥ 30), and age-related risk (e.g., for women ≥ 65; men ≥ 80). Additionally, we identified Veterans with a n Osteoporosis Self-Assessment Tool [17] score of ≤ 1 and those with evidence of exposure to chronic glucocorticoid therapy (e.g., prednisone), androgen deprivation therapy (e.g., leuprolide), or aromatase inhibitors (e.g., anastrozole) [18–21]. An algorithm reflecting these criteria is applied to data from the VHA Corporate Data Warehouse to identify Veterans in need of evaluation. Identified Veterans are contacted by US mail to notify them of this risk and the BHT clinic. The outreach mailing includes a letter highlighting osteoporosis risk and explaining the role of DXA, a fracture risk questionnaire, and a form to return indicating their interest in clinic enrollment (see online resource 1 for outreach packet.) Veterans who return the form indicating interest in enrollment are contacted by the BHT RNs, who review and document fracture risk questionnaire elements in the EHR and then place orders for physical therapy, falls assessment (i.e., occupational therapy), and DXA as appropriate. After DXA are completed, a nurse reviews the DXA report and calculates FRAX. Veterans with low risk osteopenia and normal bone density are contacted by mail with their results and recommendations for repeat DXA. Veterans with a radiologic diagnosis of osteoporosis or FRAX-based risk (i.e., major osteoporotic fracture risk of ≥ 20% or hip fracture risk of ≥ 3%) are stratified as “high risk” and triaged to an APP for new patient evaluation and treatment. Those Veterans meeting established criteria for osteoporosis (either by DXA or by prior fragility fracture) and high-risk osteopenia are counseled by phone on prescription therapy to reduce fracture risk. All Veterans with high fracture risk are referred for preliminary laboratory evaluation for secondary causes of osteoporosis and to assess eligibility for prescription osteoporosis therapies. A majority of medications are delivered directly to Veterans’ home by US mail. Adherence monitored varies by medication: follow-up for oral bisphosphonate therapy is completed at 3 and 12 months; for IV zoledronic acid at 1 and 12 months; and for denosumab and teriparatide every 3 months. All aspects of patient care delivery are documented in the VA electronic health record using note templates with embedded structured data labels. This supports a customized information management system developed to bolster point of care decision making.

## Process evaluation

To assess BHT feasibility, we conducted a multi-method process evaluation. Descriptive analyses were conducted for the primary and secondary outcomes using data from the VA Corporate Data Warehouse. In parallel, we collected qualitative data to understand implementation barriers. Qualitative design was informed by the Promoting Action on Research Implementation in Health Services (PARIHS) Framework [22]. The PARIHS Framework is an approach to designing an effective implementation plan as well as evaluating the effectiveness of implementation for task-oriented practice change. According to PARIHS, successful implementation is a product of ensuring that the innovation is derived from robust *evidence*, is conducted in a clinical *context* that is equipped for and open to change and uses evidence-based approaches to *facilitate* change and adoption. For this process evaluation, we measured successful implementation using clinical encounter data, and qualitative methods were used to characterize evidence, context, and facilitation. The strength of evidence was demonstrated by reported confidence in patient selection, clinical evaluation, treatment criteria, and care processes. The context for organizational readiness was examined in examples of technological, spatial, geographic, financial, human resource, administrative, and leadership factors limiting or enhancing BHT care processes. Facilitation effectiveness was identified in instances reflecting clinic buy-in, technical or instrumental support, and team functioning.

Qualitative data include in-person interviews and observations of clinical workflow and new site implementation discussions, collected by the evaluation team during 3 site visits (two at the BHT hub and one during new site onboarding). All staff who were potentially involved or impacted by the activities of the BHT were invited to participate, including administrators responsible for assisting Veterans in obtaining care in the community, hospital administrative staff and leaders, and clinical stakeholders such as primary care providers, radiologists, pharmacists, physical therapists, and occupational therapists. Participants were recruited by e-mail prior to the visit, with a letter describing the evaluation’s purpose and a document containing the elements of informed consent. Staff who could not be contacted via e-mail were debriefed by the evaluation team during the site visit. Data were initially collected as handwritten field notes recorded on site, which were then elaborated into typewritten narrative documents. For observations for which there were multiple evaluation team members recording field notes, each person independently drafted narrative documents and then collaborated to integrate the notes into a single observation record. For interview notes, two team m embers recorded notes: the primary note taker was not leading the interview; the interviewer served as a secondary note taker. The primary note taker drafted an initial interview summary, which was reviewed and edited by the secondary note taker using tracked changes. The primary note taker then reviewed and finalized the interview narrative. Evidence and facilitation data were identified in documents produced by BHT during usual implementation activities as well as field notes recorded by the evaluation team during interviews and observations. Contextual data were identified from field notes recorded during stakeholder interviews and observation of BHT implementation activities. Additionally, field notes documenting aspects of the built environment (e.g., workspace, signage, equipment) informed aspects of the context domain. After initial categorization, the evaluation team as a whole reviewed and refined concepts a cross the three PARIHS domains to reduce redundancies and ensure consensus.

## Results

### Primary and secondary process outcomes

Over the 15-month period, 4500 rural Veterans at risk for osteoporosis were contacted by m ail to inform them of their fracture risk and invite them to receive care through the BHT. See Table 1 for data summarizing patient engagement with screening, diagnosis, and treatment with BHT by gender. The majority of Veterans contacted were m en. Response rates by gender were similar. Approximately 64% of Veterans did not respond to the letter, 12% contact the clinic to decline care, and the remainder (24%) completed DXA screening. Of those who completed screening, 37% had normal bone density, 48% had osteopenia, and 15% had osteoporosis by DXA or by clinical fracture. For those Veterans with osteoporosis by clinical fracture, the majority (68%) had osteopenia or normal bone density, while the remainder (32%) had osteoporosis by DXA. There were 338 (31%) Veterans who met indications for prescription therapy and 306 (90 %) of these eligible Veterans initiated prescription medication.

**Table 1:** BHT enrollment, diagnosis, and prescription therapy table

	Overall		Women		Men	
	*n*	%	*n*	%	*n*	%
	4500	100.00	292	6.49	4208	93.51
Engagement with screening						
Declined	531	11.80	60	20.55	471	11.19
No response	2888	64.18	165	56.51	2723	64.71
Completed DXA	1081	24.02	67	22.95	1014	24.10
Diagnosis^a^						
Normal bone density	399	36.54	14	20.90	385	37.56
Osteopenia low risk	324	29.67	39	58.21	285	27.80
Osteopenia high risk	203	18.59	3	4.48	200	19.51
Osteoporosis by DXA	132	12.09	11	16.42	121	11.80
Osteoporosis by clinical fracture history	34	3.11	0	0.00	34	3.32
Medication indicated	338	31.27	14	20.90	324	31.95
Initiated or maintained medication	306	90.53	12	85.71	294	90.74
Refused medication	32	9.47	2	14.29	30	9.26

^a^Veterans can be diagnosed with osteoporosis by DXA and osteoporosis by clinical fracture at the spine or hip

### Implementation evaluation: context, evidence, and facilitation

Qualitative implementation data were collected over an 18-month period from 30 subjects and include 19 observations of clinic implementation activities and 12 informal interviews. During this time, the telehealth clinic was successfully implemented at 3 sites. We examined the three PARIHS framework elements—context, evidence, and facilitation—in our qualitative data [22]. The *contextual factors* affecting implementation feasibility that we identified include a geographically dispersed target population, patient characteristics, stakeholder engagement, and resource availability within the larger healthcare system. The perceived strength of the *evidence* supporting the delivery model was evaluated according to implementation complexity, adequacy of the data infrastructure for care management, the evidence base underlying patient selection and treatment, and perceived relative advantage of the BHT model over current models of care. Both the formal *facilitators* of team leadership and team members who acted as ad hoc, informal facilitators were seen to support BHT implementation. Facilitators provided assistance to achieve tasks and reach goals, which empowered team m embers to develop their own skills and abilities. A brief description of PARIHS elements and their interrelationship is provided below to illustrate the implementation experience (see Table 2) [22].

**Table 2:** Rural BHT implementation barriers and facilitators within the PARIHS framework

PARIHS framework’s elements of successful implementation		
Context	Evidence	Facilitation
Geographic service area Population characteristics Clinical stakeholders Resource availability	Implementation complexity Data infrastructure Evidence base for care delivery Stakeholder buy-in	Formal and informal facilitators Responsiveness Team-led initiative

Contextual factors of rural geography and patient characteristics shaped the BHT’s care delivery design and complicated implementation. The BHT service area encompasses the northern Rocky Mountains, including Utah, Montana, Wyoming, western Colorado, and eastern Nevada. This region’s rugged terrain, harsh seasons, long distances between population centers, and low population density impact the potential for care delivery. BHT mail and phone outreach efforts are limited by patient access to cellular, internet, and mail service. DXA is only available in urban centers, posing a barrier for patients who must travel long distances on roads which may be impassable in winter. Eligible patients are largely older adults with typical age-associated visual and hearing acuity limitations which affect phone and online communication as well as ability to travel. Eligible patients are almost all men, a population who may not believe themselves to be vulnerable to osteoporosis [23, 24].

In their clinic design and implementation plan, the BHT leveraged infrastructure and personnel strengths to overcome contextual factors they could not change. The complexity of clinic implementation was lessened, however, by characteristics shared a cross sites (e.g., a comprehensive national EHR accessible to all team m embers; national mail order pharmacy; and few to no financial barriers to ordering DXA or medications). For example, BHT was able to use a n informatics approach to identify patients across their catchment area and coordinate their evaluation and treatment rather than rely on a process of care that requires personnel or a fracture to trigger patient identification. The team developed an information display, driven by the data from the EHR, that the team’s APPs used to track patients. BHT adopted an evidence-based, validated risk indicator (i.e., OST) to identify patients at risk of OP in need of evaluation. Beyond informatics, the BHT worked to anticipate potential challenges of their context.

For example, to alleviate patients’ potential travel challenges due to adverse winter weather, the PSAs conducted initial outreach to patients in non-winter months. The BHT designed a system of evaluation, treatment recommendations, and medication delivery that could be conducted remotely, and facilitated patient access to DXA by arranging for testing in coordination with other VHA appointments or at a site closer to the Veteran’s home. The BHT was responsive to emergent challenges, a flexibility encouraged by facilitators. The PSAs conducted initial outreach to at-risk patients through mail, as they discovered that hearing challenges and poor cellular reception could make initial phone contact difficult.

Clinic implementation was complex and required significant personnel and infrastructure. The BHT comprised 8 members with varying clinical and administrative roles, making personnel a primary resource. Team leaders worked to ensure team m embers met the requirements of their roles. Depending on their role, clinicians needed expertise in informatics, OP evaluation and management, scheduling, administrative office skills, and the care processes of organizational stakeholders with whom they coordinated work (e.g., radiology for DXA). While the underlying data structure was observed to be sufficient for identification of patients at risk in need of care, the clinical team invested significant resources developing data management tools (i.e., a clinical dashboard) to facilitate care processes. The team also had attendant infrastructure needs, such as space and communication technologies. The formal facilitators on the team acted as intermediaries for the team to both ensure the team’s needs were being met and to garner support from the local departments impacted by the care processes of the BHT. For example, the facilitators worked diligently to acquire office space that enabled the team to more efficiently provide care.

Communication between facilitators and the team was critical to successful implementation. Implementation was positively impacted when formal facilitators relayed information or changes that impacted team members’ work and when direction was given to the team concerning within-team changes and transitions, such as when staff turnover occurred. Weekly meetings with team m embers aided in the identification of barriers, development of solutions, refinement of roles and care processes, responsive and flexible goal setting, and the celebration of achievements. Team members also stepped into ad hoc, informal facilitation roles that helped m embers grow individually and within their roles on the team. Informal facilitation allowed team members to trial new processes that gave them the ability to not only test out their skills, but that also lead to the development of new resources for the team to use in patient care. For example, the PSAs drew on their own experience as VA users to revise the initial outreach letter for clarity. Team member were engaged in in decisions that impacted them, including changes to role descriptions and hiring decisions. Informal facilitators on the team were involved in efforts to provide both educational materials and guides for each role. These efforts provided team m embers with the opportunity to improve their knowledge concerning OP and to develop their skills through the use of task-based tutorials.

Outside the BHT, bone health care delivery involves multiple external clinical stakeholders, including ambulatory and specialty care leadership, primary care, endocrinology, and/or rheumatology, radiology, pharmacy, and physical and occupational therapy. The BHT had to attend to localized conditions and protocols, such as service availability, care coordination agreements, or protocols with non-VHA care. One unique aspect of VA care delivery in rural area s is Veterans’ ability to see non-VHA providers a t the VHA’s expense which has the benefit of potentially reducing patient travel burden, but also can create scheduling and information sharing challenges. As a hub, the BHT adapted their care processes to stakeholders across multiple sites, each with different workflows and organizational cultures. As BHT was implemented at new locations, facilitators committed to in-person site visits which smoothed the team’s way forward at each site. The team established open and ongoing communication with site providers, especially in primary care, to facilitate buy-in without being burdensome. Stakeholders external to the clinic expressed confidence in the team’s expertise and accepted treatment decisions. External stakeholders across disparate roles (e.g., clinical, administrative, leadership, etc.) expressed enthusiasm for the care delivery model and its superiority to usual care processes, particularly regarding the clinic’s emphasis on minimizing workload sent to primary care. Stakeholders viewed BHT’s ownership of OP care (i.e., identification, evaluation, treatment, and adherence monitoring), and an advantage to other initiatives which rely on clinical reminders or simply identify additional work to be performed by primary care providers.

## Discussion

A clinical and an evaluation team collaborated to conduct a process evaluation of a telehealth clinic implemented to identify and treat Veterans at risk of osteoporotic fracture. In doing so, we demonstrate both the feasibility of delivering care using this model and the potential for model refinement. In this study, 24% of Veterans who were offered DXA completed the screening. This finding is similar to what has been reported in small randomized trials evaluating primary prevention in osteoporosis utilizing mail-out invitations to participate in DXA screening, with DXA completion ranging from 13 to 28%, though not all studies have shown improvement beyond usual care [25–27]. In this cohort of BHT patients, one third of the Veterans completing screening were candidates for prescription therapy to reduce fracture risk, and most (91%) initiated treatment. This finding represents a high uptake of prescription therapy in contrast to the rates of treatment uptake observed in the primary prevention studies utilizing mail-out screening invitations [25–27] ranging between 3 and 9% of those eligible. Our study found that contextual factors of rural geography and characteristics of rural Veterans at risk for osteoporosis were barriers to DXA screening. This finding is consistent with others who have shown that distance to DXA screening, older age, multimorbidity, and socioeconomic factors are barriers to population-based DXA screening [28]. Our BHT patient population was predominantly male (93%), a population found to have low perceived susceptibility to and knowledge of osteoporosis [12], which may influence osteoporosis screening uptake. We found the complexity of clinic implementation, especially the high demand for personnel to support care processes, to be the primary evidence barrier in need of refinement. Though a cost-effectiveness analysis of a prior pilot of this care model found it to be cost-effective [29], significant infrastructural resources could diminish feasibility without considerable leadership buy-in. There were many implementation facilitators of the BHT care model that spanned context, evidence, and facilitation. The contextual factors of clinical stakeholder engagement and availability of funding to support BHT operations provided a counterweight to the inflexible contextual barriers of rural geography and patient features. In addition to leveraging data infrastructure and guideline-based clinical protocols, external stakeholder buy-in was a crucial evidence facilitator to program implementation and enabled expedited clinical operations.

Facilitation factors enabling implementation success included formal facilitators who prioritized the training of informal facilitators within the multidisciplinary BHT, allowing team members to develop new processes, skills, and resources that furthered rural Veteran care, which resulted in a largely team-lead initiative.

A growing quality gap in osteoporosis care has been identified in recent years with insufficient evaluation and declining treatment of at-risk patients [9, 30, 31], despite increasing fragility fracture incidence [32]. A variety of osteoporosis care delivery models ranging from less effective patient and/or provider education-only initiatives [33–36] to more effective multifaceted and coordinator-based programs [37–39] have reported improvement in osteoporosis screening and/or treatment after a fracture. However, care delivery models that address primary prevention of osteoporotic fracture are limited [25, 40–42], especially in men, and generally rely on primary care providers to implement risk identification, screening, and management. A recent meta-analysis of interventions to improve osteoporosis care found that interventions targeting the health system were more effective than those focused on healthcare providers or patients [43]. Moreover, challenges faced by primary care, especially rural care providers (e.g., provider shortages, large panel sizes, and time barriers), combined with a growing population at risk for osteoporotic fractures, will require innovative and team-based approaches to population bone health [44]. The BHT model leverages informatics-driven risk identification, an integrated EHR, low cost to Veterans mail-order pharmacy, the ability of clinical pharmacists to work as advanced practice providers within the VHA, and low-to-no Veterans cost DXA to coordinate bone health care on behalf of primary care, circumventing the customary reliance on primary care for screening and treatment.

It is important to note that our study has several limitations. First, given the observation period, we did not measure the impact of screening or treatment on fracture rates, similar to other studies evaluating bone health outcomes of screening and treatment initiation [13, 22]. Because the current analysis was designed to ascertain program feasibility, we did not statistically examine the factors influencing rates of enrollment or treatment, adherence rates among those who initiated therapy, or analyze process measures longitudinally. These analyses, as well as a qualitative study of BHT patient experience, will be pursued in future research. Finally, VHA represents a unique environment, and this model of care may not be transferable to or feasible in other health care environments. Despite these limitations, the availability of implementation resources for this program allowed us to identify barriers to care delivery beyond those associated with resource constraints.

A recent report from the Federal Partners Meeting of the National Institutes of Health Pathways to Prevention Workshop highlighted the need for pragmatic randomized, controlled trials to study the efficacy of various strategies to osteoporosis management, in addition to understanding the factors that influence patient decision-making with osteoporosis treatment [45]. They called for research studies that determine the context most conducive to shared decision-making in order to surmount barriers related to patients and providers and emphasized the value of pilot studies and smaller implementation studies to inform future pragmatic trials. Our study examined both the feasibility system for providing virtual population bone health and the implementation process for delivering these services to rural Veterans in the Rocky Mountain West. Those interested in implementation of a similar process to provide population bone health may find their efforts most facilitated by the development of systems that reduce complete dependence on primary care or other providers for risk identification, screening, and treatment. Other important process enablers include acquisition of buy-in from all stakeholders, use of guideline-based protocols that permit clinical pharmacists and nurses to operate at the highest levels of their licenses, and the development of health information technology tools to allow streamlining of data for operational and clinical use. Aspects of this model in need of refinement include restructuring clinic processes to reduce personnel support, simplification of clinical note templates, and enhanced communication with patients to improve DXA acceptance (see online resource 2 for a detailed list of available BHT resources).

## Conclusion

In this analysis, context was employed to understand both the a priori, external-to-the-intervention, and often immovable factors limiting or enhancing BHT care processes, and the BHT’s ability to address or leverage those factors. We found the BHT model to be a feasible approach to population bone health with similar engagement to other population health programs, but with high prescription medication uptake in Veterans meeting indications for treatment. Current evaluation of patient-level data is underway to understand Veteran decisions in relation to choosing or declining osteoporosis screening and initiation of prescription therapies. Future research should evaluate the implementation factors associated with the most effective multi-faceted osteoporosis interventions across a variety of health care contexts, to guide population bone health initiatives.
